# Tibial to ulnar nerve amplitude ratio as a marker of length-dependent neuropathy

**DOI:** 10.1016/j.cnp.2025.10.006

**Published:** 2025-10-25

**Authors:** Chikashi Yano, Tomonori Nakamura, Masahiro Ando, Yujiro Higuchi, Jun-Hui Yuan, Akiko Yoshimura, Takahiro Hobara, Fumikazu Kojima, Yu Hiramatsu, Satoshi Nozuma, Yusuke Sakiyama, Akihiro Hashiguchi, Raymond L. Rosales, Kimiyoshi Arimura, Hiroshi Takashima

**Affiliations:** a Department of Neurology and Geriatrics, Kagoshima University Graduate School of Medical and Dental Sciences, Kagoshima, Japan; b Department of Neuroscience and Research Center for Health Sciences, University of Santo Tomas Faculty of Medicine and Surgery, Manila, Philippines; c Okatsu Neurology and Rehabilitation Hospital, Kagoshima, Japan

**Keywords:** Electrophysiology, CMT, Differential diagnosis, Nerve conduction study, Neuropathies, Acquired

## Abstract

**Objective:**

To evaluate the utility of nerve conduction studies as a marker of length-dependent neuropathy.

**Methods:**

We conducted a retrospective study of 44 chronic inflammatory demyelinating polyradiculoneuropathy (CIDP) patients and 365 genetically confirmed Charcot-Marie-Tooth disease (CMT) patients, including those with *PMP22* duplications or mutations in *GJB1*, *MFN2*, *MPZ*, and *MME*. Nerve conduction study parameters were compared, with subgroup analyses of CIDP-mimicking CMT (genetically confirmed CMT with a prior clinical diagnosis of CIDP) and gene-based classifications. Receiver operating characteristic (ROC) analysis assessed the sensitivity and specificity of these parameters.

**Results:**

The tibial to ulnar nerve distal compound muscle action potential (T/U CMAP) amplitude ratio was significantly higher in CIDP patients compared to those with genetically confirmed CMT, CIDP-mimicking CMT, and gene-based subgroups. This ratio yielded the highest area under the curve (AUC: 0.95) among all evaluated parameters, with a cutoff value of 0.385 demonstrating high diagnostic sensitivity (95.5%) and specificity (85.5%). In CIDP-mimicking CMT group, a similar sensitivity and specificity were observed.

**Conclusions:**

The T/U CMAP amplitude ratio is a simple, robust electrophysiological index of length-dependent neuropathy.

**Significance:**

This marker offers a reliable and accessible way to distinguish between acquired and inherited neuropathies, improving diagnostic accuracy and helping prioritize genetic testing.

## Highlights

• Tibial to ulnar amplitude ratio separates acquired and inherited neuropathies.
• The ratio is higher in chronic inflammatory demyelinating polyradiculoneuropathy.
• Area under the curve 0.95 with 95.5% sensitivity and 85.5% specificity.

## 1 Introduction

Charcot-Marie-Tooth disease (CMT) and chronic inflammatory demyelinating polyradiculoneuropathy (CIDP) are distinct neurological disorders that have overlapping clinical and electrophysiological features, which often leads to diagnostic challenges (Campagnolo et al., 2020, Hauw et al., 2021). Both conditions are characterized by chronic, progressive neuropathies, typically presenting with muscle weakness, sensory loss, and impaired reflexes. Genetic testing remains the diagnostic gold standard for CMT and can resolve many uncertainties; however, it is not always immediately available, and misdiagnosis may expose patients to prolonged, unnecessary immunotherapy and healthcare burden (Bouchard et al., 1999, Hauw et al., 2021). Several factors contribute to the misdiagnosis of CMT, including the absence of family history and partial responsiveness to immunotherapy (Allen and Lewis, 2015, Ginsberg et al., 2004, Marques et al., 2010, Vaeth et al., 2021, Watanabe et al., 2002).

Historically, the presence of abnormal temporal dispersion and conduction block has been considered a key feature distinguishing CIDP from inherited neuropathies (Lewis and Sumner, 1982). The electrodiagnostic criteria for CIDP incorporate demyelinating features such as nerve conduction velocity slowing and conduction block (Van den Bergh et al., 2021). However, some CMT subtypes, such as those with *GJB1* or *MPZ* mutations, can exhibit these electrophysiological features (Ginsberg et al., 2004, Gutierrez et al., 2000, Marques et al., 2010, Michell et al., 2009, Miki et al., 2013, Tabaraud et al., 1999). Misinterpretation of these findings is a factor that leads to CIDP misdiagnosis (Allen et al., 2018). Accordingly, identifying a physiology-based electrophysiological index, together with salient clinical patterns, may aid differentiation between CIDP and CMT in routine practice.

Because CMT typically shows length-dependent axonal loss, we hypothesized that the tibial to ulnar CMAP amplitude ratio would be lower in CMT than in CIDP, whereas CIDP—being an acquired, diffuse demyelinating neuropathy—would show a relatively higher ratio. (Verhamme et al., 2004) We therefore compared nerve conduction parameters between CIDP and genetically confirmed CMT, and performed subgroup analyses including CIDP-mimicking CMT (CMT cases carrying a prior clinical diagnosis of CIDP) as well as genotype-based strata.

## 2 Materials and methods

### 2.1 Inclusion and exclusion criteria of patients with CIDP or CMT

Data were retrospectively collected from patients diagnosed with CIDP at Kagoshima University Hospital and from patients with genetically confirmed CMT who were referred to our department for genetic testing between January 1990 and December 2023. The Institutional Review Board of Kagoshima University reviewed and approved the study protocol (approval number: 230266).

The CIDP diagnosis was established based on the European Academy of Neurology/Peripheral Nerve Society (EAN/PNS) criteria (Van den Bergh et al., 2021). Patients with CMT who were included in this study were genetically diagnosed using fluorescent in situ hybridization, multiplex ligation-dependent probe amplification, gene-panel or whole-exome sequencing, as previously described (Yoshimura et al., 2019).

The cohort comprised patients with the most common CMT-related gene mutations, including the *PMP22* duplication and mutations in the *GJB1*, *MFN2*, *MPZ*, or *MME* genes (Ando et al., 2022).

**Fig. 1:**
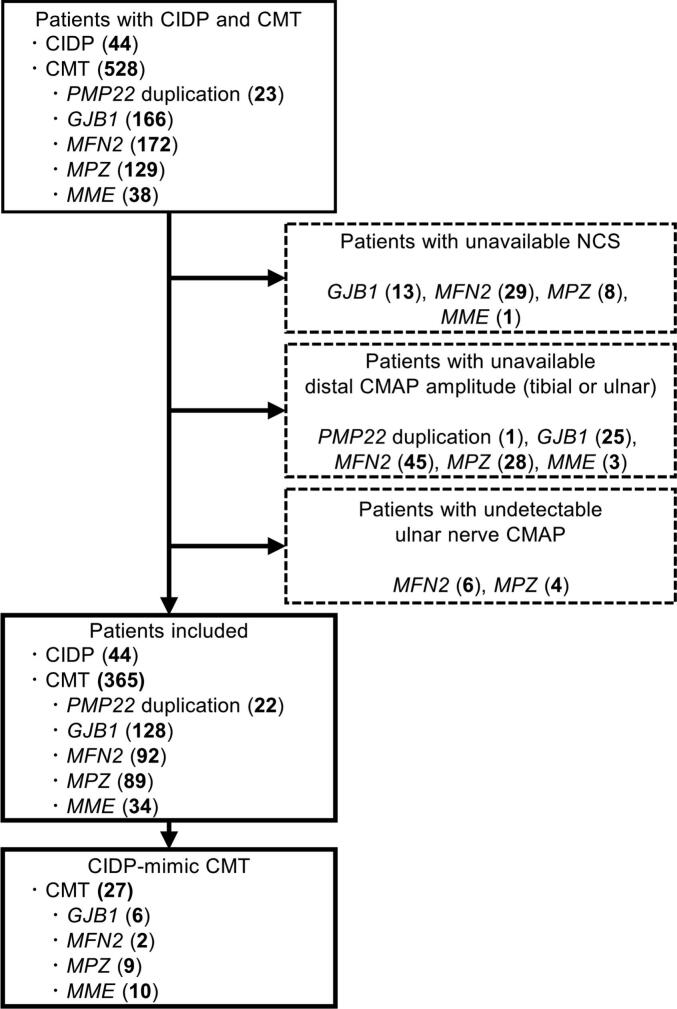
Flow of patient selection. CIDP, chronic inflammatory demyelinating polyradiculoneuropathy; CMT, Charcot-Marie-Tooth disease; NCS, nerve conduction study; CMAP, compound muscle action potential.

The inclusion criteria required the availability of nerve conduction study (NCS) data for both the ulnar and tibial nerves in all patients. Patients with undetectable ulnar nerve CMAP or those with other primary peripheral nerve disorders, such as severe diabetes mellitus or compressive/traumatic radiculo-plexo neuropathies, were excluded from the study (Fig. 1).

### 2.2 Clinical study

The collected clinical data included sex, family history, consanguinity, age at disease onset, age at the time of NCS, and NCS parameters. CIDP-mimicking CMT was defined as genetically confirmed CMT in patients who had previously received a clinical diagnosis of CIDP before genetic testing. Use of immunotherapy was recorded but was not required for inclusion.

### 2.3 NCS

NCSs were performed using established techniques (hinged on the normative data from electrodiagnostic (EDX)/EDX laboratories) and commercially available EDX equipment (Kimura, 2001). During NCS, the patient’s skin temperature was maintained above 33 °C in the upper limb and 30 °C in the lower limb. CMAP amplitude was measured from baseline to the most negative peak. Meanwhile, CMAP duration was defined as the time interval between the initial deflection from baseline and the return to baseline of the last negative peak.

To compare the CMAP amplitudes between the upper and lower limb, the ulnar nerve was selected for the upper limb, as the median nerve can be affected by carpal tunnel syndrome. Further, the tibial nerve was chosen for the lower limb, as the fibular nerve is commonly impaired in Japanese individuals, even in healthy populations (Kishi et al., 2017, Kishimoto et al., 2021).

The tibial to ulnar (T/U) CMAP amplitude ratio was determined by dividing the distal CMAP amplitude of the tibial nerve by that of the ulnar nerve. The terminal latency index (TLI) was calculated using the following formula: TLI = terminal distance (mm)/(distal latency (ms) × conduction velocity (m/s)) (Attarian et al., 2001). To minimize the influence of compressive neuropathies, such as cubital tunnel syndrome, proximal-to-distal (p/d) CMAP amplitude, and duration ratios for the ulnar nerve were calculated using below-elbow measurements. For the tibial nerve, measurements were taken from the popliteal fossa and the ankle.

### 2.4 Statistical analyses

Data were presented as median values with interquartile ranges (IQRs). Between-group comparisons for quantitative and qualitative variables were performed using the two-sided Wilcoxon rank-sum test and Fisher’s exact test, respectively.

The prespecified primary comparison was CIDP vs pooled, genetically confirmed CMT. For this single comparison, results were interpreted conservatively using a significance threshold of *p* < 0.001. For the five prespecified genotype-level secondary comparisons (CIDP vs each of *PMP22* duplication, *GJB1*, *MFN2*, *MPZ*, and *MME*), multiplicity was controlled using the Benjamini–Hochberg procedure with a false discovery rate (FDR) of 5 %; adjusted *q* values are reported, with *q* < 0.05 considered statistically significant.

Receiver operating characteristic (ROC) analysis was conducted to evaluate the diagnostic performance of each parameter in determining CIDP. The optimal cutoff value was defined as the point nearest to the upper left corner of the ROC curve. Pearson’s correlation coefficient was calculated to assess the relationship between disease duration and T/U CMAP amplitude ratio. All statistical analyses were conducted using RStudio (version 4.2.1).

When CMAP or sensory nerve action potential (SNAP) amplitudes were not detectable, values were treated as 0 for amplitude-based analyses. For parameters required a measurable response—CMAP duration, distal latency, TLI, and conduction velocity—observations with absent responses were excluded. For the T/U ratio specifically, cases with ulnar dCMAP = 0 were excluded (n = 10) because the ratio is undefined, whereas cases with tibial dCMAP = 0 but a measurable ulnar dCMAP were assigned a T/U ratio of 0 by definition.

## 3 Results

### 3.1 Selection of patients

In total, 572 patients, comprising 44 with CIDP and 528 with genetically confirmed CMT, were initially enrolled. Of these, 51 were excluded due to the lack of NCS data, 102 due to missing distal CMAP amplitudes in either the tibial or ulnar nerve, and 10 due to undetectable CMAP in the ulnar nerve (Fig. 1). Among the 10 patients with undetectable CMAP, 6 had undetectable responses in the median and tibial nerves, and 2 had extremely low median nerve amplitudes (in the 0.1-mV range). Of the remaining two patients, one had undetectable median and ulnar nerves but detectable tibial nerve responses. Meanwhile, the other had undetectable ulnar and tibial nerves but a detectable median nerve response. Consequently, 44 patients with CIDP—including typical CIDP (n = 29), distal CIDP (n = 8), and multifocal CIDP (n = 7)—and 365 patients with CMT—including individuals with *PMP22* duplication (n = 22) and *GJB1* (n = 128), *MFN2* (n = 92), *MPZ* (n = 89), and *MME* (n = 34) mutations—were included in the analyses (Table 1).

**Table 1:** Clinical and genetic summary of all patients.

	CIDP (n = 44)		CMT (n = 365)		*p* value	CIDP-mimic CMT (n = 27)		*p* value
Sex (M:F)	29:15		234:131		0.869	17:10		0.804
Age	60 (35.75–66.25)		47 (30–60)		0.007	64 (50–68.5)		0.243
Age of onset	58.5 (34.75–65)		18 (6–44)		<0.001	48 (39–54)		0.384
Disease duration (years)	0.5 (0–2) (n = 44)		15 (6–30) (n = 362)		<0.001	7 (3.5–13) (n = 27)		<0.001
Family history or consanguinity	0 % (0/44)		60.3 % (220/365)		<0.001	59.3 % (16/27)		<0.001
typical CIDP	29	66 %						
distal CIDP	8	18 %						
multifocal CIDP	7	16 %						
*PMP22* duplication (%)			22	6 %		0	0 %	
*GJB1* (%)			128	35 %		6	22 %	
*MFN2* (%)			92	25 %		2	7 %	
*MPZ* (%)			89	24 %		9	33 %	
*MME* (%)			34	9 %		10	37 %	

CIDP, chronic inflammatory demyelinating polyradiculoneuropathy; CMT, Charcot-Marie-Tooth disease; M, male; F, female.

To quantify genotype-specific missingness, we tabulated absent distal responses within the CMT cohort in whom motor NCS were attempted (n = 375; denominators per genotype shown). Ulnar dCMAP was absent in *MFN2* 6/98 (6.1 %) and *MPZ* 4/93 (4.3 %), and in 0/22 *PMP22* duplication, 0/128 *GJB1*, and 0/34 *MME*. By contrast, tibial dCMAP was more frequently absent: *PMP22* duplication 6/22 (27.3 %), *GJB1* 33/128 (25.8 %), *MFN2* 36/98 (36.7 %), *MPZ* 34/93 (36.6 %), and *MME* 18/34 (52.9 %). As prespecified, observations with ulnar dCMAP = 0 were excluded from T/U-ratio analyses (the ratio is undefined), whereas tibial-absent observations were retained for amplitude-based summaries and assigned a T/U ratio of 0 when the ulnar dCMAP was measurable.

Of the 365 CMT cases analyzed, 27 (7.4 %) had a prior diagnosis of CIDP before genetic confirmation. Among them, 23 received immunotherapy (IVIG in all; corticosteroids in 5, dexamethasone in 1, and plasma exchange in 2). The provisional CIDP diagnosis was most often based on albuminocytologic dissociation in CSF and demyelinating features on nerve conduction studies (e.g., abnormal temporal dispersion or conduction block). The identified genotypes of these cases were *MME* (n = 10), *MPZ* (n = 9), *GJB1* (n = 6), and *MFN2* (n = 2). These 27 patients constitute the “CIDP-mimicking CMT” group (see Supplementary Table 1).

### 3.2 Comparison between CIDP and genetically confirmed CMT

#### 3.2.1 Clinical comparisons

Patients with CIDP and genetically confirmed CMT did not significantly differ in terms of sex or age. However, patients with CIDP had a higher age at disease onset than those with CMT (58.5 vs. 18.0 years; *p* < 0.001). Patients with CIDP had a significantly shorter disease duration than those with CMT (0.5 vs. 15 years; *p* < 0.001). Family history or consanguinity was not observed in patients with CIDP, whereas it was present in 60.3 % of those with CMT (*p* < 0.001).

#### 3.2.2 NCS analyses

**Fig. 2:**
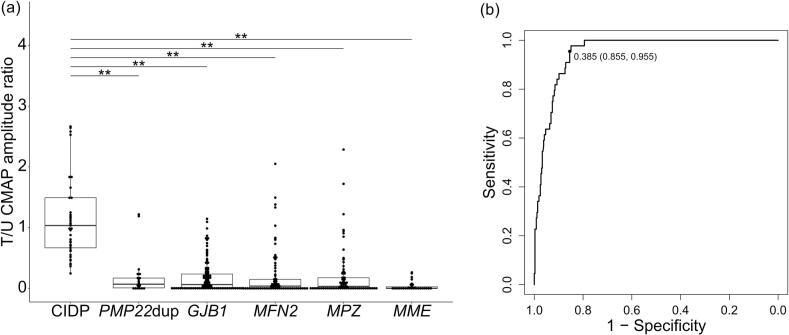
Tibial to ulnar nerve CMAP amplitude ratio in CIDP and CMT. (a) Patients with CIDP had a higher T/U CMAP amplitude ratio than those with genetically diagnosed CMT. There were seven outliers that fall outside the plotted range: 3.35, 3.59, 3.93, 5.53, 7.82, and 7.83 in CIDP and 6.00 in *GJB1* (** *q* < 0.001). (b) Receiver operating characteristic curve analysis was performed to distinguish CIDP from genetically confirmed CMT. The area under the curve was 0.95, with a sensitivity of 95.5 % and a specificity of 85.5 %. CMAP, compound muscle action potential; CIDP, chronic inflammatory demyelinating polyradiculoneuropathy; CMT, Charcot-Marie-Tooth disease; T/U CMAP amplitude ratio, tibial nerve-to-ulnar nerve compound muscle action potential amplitude ratio; PMP22dup, *PMP22* duplication.

Patients with CIDP had a significantly higher T/U CMAP amplitude ratio than those with CMT (1.07 vs. 0.04; *p* < 0.001). In gene-based subgroup analyses (*PMP22* duplication, *GJB1*, *MFN2*, *MPZ*, *MME*), the T/U CMAP amplitude ratio was higher in CIDP than in each CMT genotype. These differences remained significant after Benjamini–Hochberg correction for the five planned comparisons (all *q* < 0.001), with large effect sizes (Cliff’s delta 0.886–0.997) (Fig. 2A). The relationship between the T/U CMAP amplitude ratio and disease duration is shown in Supplementary Fig. 1 (Fig. S1). In both the CIDP and CMT groups, no significant correlation was observed between the T/U CMAP amplitude ratio and disease duration (*r* = 0.018 and –0.06, respectively).

Compared with patients with CMT, those with CIDP had significantly prolonged distal and proximal CMAP durations and distal latencies in the ulnar nerve, remarkably higher distal and proximal CMAP amplitudes, prolonged distal and proximal CMAP durations, faster motor nerve conduction velocities in the tibial nerve, significantly higher SNAP amplitudes, and faster sensory nerve conduction velocities in the sural nerve (Table 2).

**Table 2:** Nerve conduction studies with statistical analyses of CIDP and CMT.

	CIDP		CMT				CIDP-mimicking CMT			
	Median	n = 44	Median	n = 365	*p* value	AUC	Median	n = 27	*p* value	AUC
T/U CMAP amplitude ratio	1.07 (0.69–1.83)	44	0.04 (0–0.18)	365	<0.001	0.95	0.02 (0.00–0.26)	27	<0.001	0.97
Ulnar nerve										
Distal CMAP amplitude (mV)	4.60 (3.3–6.4)	44	5.10 (3.01–7.6)	365	0.34	0.54	6.10 (3.65–7.6)	27	0.100	0.62
Proximal CMAP amplitude (mV)	3.22 (1.87–4.6)	44	3.89 (2.3–6.18)	333	0.072	0.58	4.20 (2.75–6.07)	26	0.072	0.63
CMAP amplitude ratio (p/d)	0.71 (0.6–0.85)	44	0.83 (0.65–0.93)	333	0.012	0.62	0.73 (0.61–0.95)	26	0.466	0.55
Distal CMAP duration (ms)	7.30 (6.12–9.57)	34	6.00 (5.3–6.9)	237	<0.001	0.72	5.8 (5.5–6.4)	21	0.002	0.75
Proximal CMAP duration (ms)	8.45 (6.88–10.1)	33	6.5 (5.7–7.8)	234	<0.001	0.71	6.37 (5.76–7.32)	20	0.003	0.75
CMAP duration ratio (p/d)	1.11 (1.02–1.26)	33	1.09 (1.02–1.19)	234	0.45	0.54	1.11 (1.06–1.23)	20	0.942	0.51
Distal latency (ms)	4.75 (3.7–5.6)	44	3.5 (2.9–4.55)	363	<0.001	0.69	3.3 (2.9–3.89)	27	<0.001	0.79
TLI	0.50 (0.42–0.57)	44	0.46 (0.4–0.55)	231	0.55	0.53	0.46 (0.41–0.49)	19	0.163	0.61
MCV (m/s)	36.45 (28.58–45.7)	44	39.90 (31.5–50.6)	361	0.15	0.57	43.65 (35.97–51.6)	26	0.050	0.64
										
Tibial nerve										
Distal CMAP amplitude (mV)	4.65 (2.9–7.67)	44	0.20 (0–0.8)	365	<0.001	0.93	0.121 (0–1.46)	27	<0.001	0.92
Proximal CMAP amplitude (mV)	2.60 (1.48–4.32)	44	0.09 (0–0.4)	319	<0.001	0.92	0.099 (0–0.66)	24	<0.001	0.92
CMAP amplitude ratio (p/d)	0.61 (0.45–0.77)	44	0.63 (0.37–0.88)	319	0.51	0.53	0.633 (0.31–0.87)	24	0.901	0.51
Distal CMAP duration (ms)	8.50 (6.5–11.2)	34	5.20 (3.7–7.3)	152	<0.001	0.80	5.9 (4.5–7.8)	13	0.005	0.77
Proximal CMAP duration (ms)	10.00 (8.3–11.85)	34	5.75 (4–9.75)	136	<0.001	0.72	7.7 (4.65–10.1)	11	0.075	0.68
CMAP duration ratio (p/d)	1.13 (1.05–1.22)	34	1.10 (0.96–1.35)	136	0.76	0.52	1.04 (0.98–1.36)	11	0.593	0.56
Distal latency (ms)	5.24 (4.7–7.58)	44	6.15 (4.9–8.85)	247	0.049	0.59	5.15 (5.00–6.42)	18	0.786	0.48
TLI	0.40 (0.34–0.45)	44	0.42 (0.31–0.54)	134	0.34	0.55	0.573 (0.34–0.64)	8	0.1083	0.68
MCV (m/s)	36.00 (30.23–42.57)	44	29.85 (22.95–35.85)	224	<0.001	0.70	28.00 (22.2–29.8)	15	0.002	0.78
										
Sural nerve										
SNAP amplitude (μV)	5.10 (1.09–11.8)	43	0.00 (0–2.08)	342	<0.001	0.79	0.00 (0–1.7)	26	<0.001	0.79
SCV (m/s)	44.60 (38.7–49.8)	35	35.95 (31.58–44.6)	124	<0.001	0.73	37.3 (34.25–40.3)	11	0.006	0.78

Reference value: ulnar nerve distal CMAP amplitude, > 6.0 mV; ulnar nerve distal latency, < 3.6 ms; ulnar nerve MCV, > 50.1 m/s; tibial nerve distal CMAP amplitude, > 4.4 mV; tibial nerve distal latency, < 5.7 ms; tibial nerve MCV, > 41.7 m/s; sural nerve SNAP amplitude, > 5.0 μV; sural nerve SCV, > 40.8 m/s.

CIDP, chronic inflammatory demyelinating polyradiculoneuropathy; CMT, Charcot-Marie-Tooth disease; AUC, area under the curve; T/U CMAP amplitude ratio, tibial nerve-to-ulnar nerve distal compound muscle action potential amplitude ratio; CMAP, compound muscle action potential; p/d, proximal/distal; TLI, terminal latency index; MCV, motor nerve conduction velocity; SNAP, sensory nerve action potential; SCV, sensory nerve conduction velocity.

#### 3.2.3 ROC analysis

Among the evaluated parameters when classifying CIDP as “positive,” the T/U CMAP amplitude ratio had the highest area under the curve (AUC) value at 0.95 (Table 2). At a cutoff value of 0.385, the sensitivity and specificity of the T/U CMAP amplitude ratio were 95.5 % and 85.5 %, respectively (Fig. 2B).

### 3.3 Comparison between patients with CIDP and those with CIDP-mimicking CMT

#### 3.3.1 Clinical comparisons

Patients with CIDP (n = 44) and those with CIDP-mimicking CMT (n = 27) did not differ significantly in terms of sex, age, or age at onset. Disease duration was significantly shorter in CIDP patients compared to those with CIDP-mimicking CMT. Family history or consanguinity was absent in CIDP patients but present in 59.3 % of the CIDP-mimicking CMT group (*p* < 0.001).

#### 3.3.2 NCS analyses

**Fig. 3:**
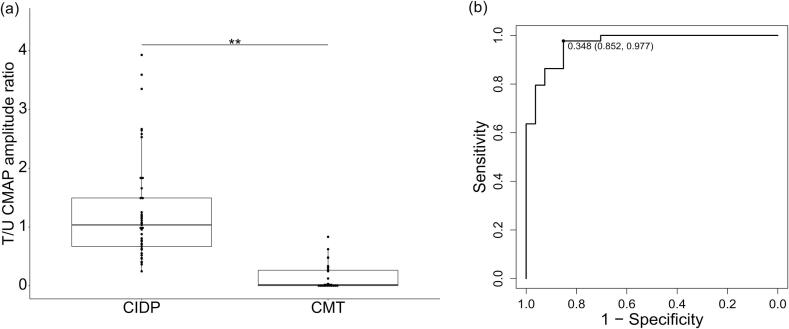
Tibial to ulnar nerve CMAP amplitude ratio in CIDP and CIDP-mimicking CMT. (a) Patients with CIDP had a higher T/U CMAP amplitude ratio than those with CIDP-mimicking CMT. There were three outliers that fall outside the plotted range: 5.53, 7.82, and 7.83 in CIDP(** *p* < 0.001). (b) A receiver operating characteristic curve analysis was performed to distinguish CIDP from CMT. The area under the curve was 0.96, with a sensitivity of 97.7 % and a specificity of 85.2 %. CMAP, compound muscle action potential; CIDP, chronic inflammatory demyelinating polyradiculoneuropathy; CMT, Charcot-Marie-Tooth disease; T/U CMAP amplitude ratio, tibial nerve-to-ulnar nerve compound muscle action potential amplitude ratio.

Patients with CIDP had a significantly higher T/U CMAP amplitude ratio than those with CIDP-mimicking CMT (Fig. 3A). Compared with the CIDP-mimicking CMT group, CIDP patients had significantly prolonged distal latencies in the ulnar nerve, markedly higher distal and proximal CMAP amplitudes in the tibial nerve, and significantly higher SNAP amplitudes in the sural nerve (Table 2).

#### 3.3.3 ROC analysis

Among the evaluated parameters for classifying CIDP, the T/U CMAP amplitude ratio demonstrated the highest AUC (0.97). Using a cutoff value of 0.348, the sensitivity and specificity of the T/U CMAP amplitude ratio were 97.7 % and 85.2 %, respectively (Fig. 3B).

### 3.4 Subgroup analysis

**Fig. 4:**
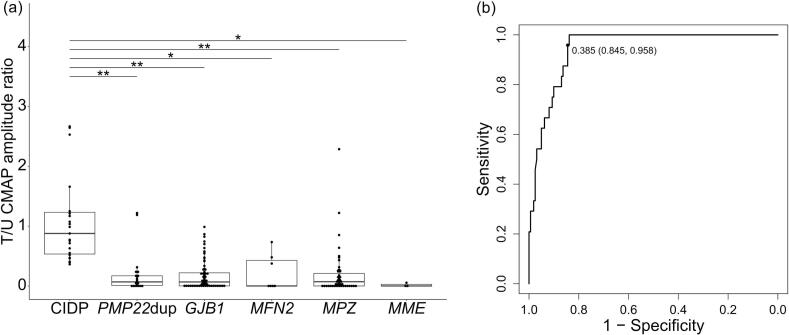
Tibial to ulnar nerve CMAP amplitude ratio in patients with demyelinating CIDP and CMT. (a) In the demyelinating subgroup, patients with CIDP had a higher T/U CMAP amplitude ratio than those with diverse CMT gene mutations. There were two outliers that fall outside the plotted range: 3.928 and 7.833 in CIDP (* *q* < 0.05, ** *q* < 0.001). (b) A receiver operating characteristic curve analysis was performed to distinguish CIDP from CMT. The area under the curve was 0.94, with a sensitivity of 95.8 % and a specificity of 84.5 %. CMAP, compound muscle action potential; CIDP, chronic inflammatory demyelinating polyradiculoneuropathy; CMT, Charcot-Marie-Tooth disease; T/U CMAP amplitude ratio, tibial nerve-to-ulnar nerve compound muscle action potential amplitude ratio; PMP22dup, *PMP22* duplication.

A subgroup analysis limited to the demyelinating type of both CIDP and CMT, which is defined by an ulnar nerve motor conduction velocity of less than 38 m/s, was conducted. This analysis enrolled 24 patients with CIDP and 161 with genetically confirmed CMT. Similar results were obtained for the T/U CMAP amplitude ratio with a cutoff value of 0.385 (AUC: 0.94, sensitivity: 95.8 %, and specificity: 84.5 %) (Table 3, Fig. 4, Fig. 4). In addition, distal CIDP and CMT were compared. Although the number of patients with distal CIDP was small (n = 8), similar results were observed (AUC: 0.96, cutoff value: 0.385, sensitivity: 100 %, and specificity: 85.5 %).

**Table 3:** Clinical and genetic summary of patients with demyelinating CIDP and CMT.

	CIDP (n = 24)		CMT (n = 161)		*p* value
Sex (M:F)	17:7		114:47		1
Age	62 (53.25–70.25)		44 (28–59)		<0.001
Age of onset	60 (44–66.75)		14 (6–35)		<0.001
Disease duration (years)	1 (0–1.25) (n = 24)		17 (6–31.2) (n = 160)		<0.001
Family history or consanguinity	0		57 % (91/161)		<0.001
Typical CIDP	16	66.7 %			
Distal CIDP	4	16.7 %			
Multifocal CIDP	4	16.7 %			
*PMP22* duplication (%)			22	14 %	
*GJB1* (%)			74	46 %	
*MFN2* (%)			7	4 %	
*MPZ* (%)			55	34 %	
*MME* (%)			3	2 %	

CIDP, chronic inflammatory demyelinating polyradiculoneuropathy; CMT, Charcot-Marie-Tooth disease; M, male; F, female.

## 4 Discussion

In this study, we present a comprehensive clinical and electrophysiological comparison between patients with CIDP and those with genetically confirmed CMT. The analyses encompassed a large cohort of patients from Japan, representing the five most common causative genes: *PMP22*, *GJB1*, *MFN2*, *MPZ*, and *MME*. Patients with CIDP and those with CMT had distinct clinical and NCS findings. However, among all the NCS parameters, the T/U CMAP amplitude ratio had the highest AUC value.

CIDP is characterized by symmetric proximal and distal muscle weakness. Meanwhile, CMT typically presents as distal muscle weakness and atrophy predominantly in the lower limbs (Van den Bergh et al., 2021, Verhamme et al., 2004). These clinical differences are caused by their distinct pathophysiological mechanisms. In CIDP, demyelination primarily affects the nerve roots and terminals (Brown and Snow, 1991). However, in CMT, axonal degeneration progresses in a length-dependent manner, starting from the distal legs. Historically, inherited neuropathies have been considered to cause uniform conduction slowing, whereas acquired neuropathies typically result in nonuniform nerve affection (Lewis and Sumner, 1982). Parameters such as the p/d CMAP amplitude ratio, p/d CMAP duration ratio, and modified F-ratio, which can quantitatively assess conduction block, temporal dispersion, and the uniformity of conduction slowing, have been reported as useful tools for differential diagnosis (Attarian et al., 2001, Kang et al., 2013, Kitaoji et al., 2023). However, a limitation of the aforementioned studies is that most patients with CMT who were included presented with CMT1A. Recent advances in genetics have revealed that patients with certain CMT types, such as those with *GJB1* or *MPZ* mutations, can also exhibit abnormal temporal dispersion or conduction block (Ginsberg et al., 2004, Gutierrez et al., 2000, Marques et al., 2010, Michell et al., 2009, Miki et al., 2013, Murphy et al., 2011, Street et al., 2002, Tabaraud et al., 1999) The utility of these parameters for differentiating CIDP from non-CMT1A genotypes remains uncertain. In view of these previous limitations in applying EDX study parameters, including late-response studies, we carefully chose other parameters of potential diagnostic practicality.

In addition, our analysis revealed that CMAP duration was significantly prolonged in both the ulnar and tibial nerves in patients with CIDP compared to those with CMT. Similarly, the distal latency of the ulnar nerve was also significantly prolonged in CIDP. These findings are consistent with the established electrophysiological characteristics of CIDP, as reflected in the diagnostic criteria, which include prolonged CMAP duration and distal latency (Van den Bergh et al., 2021). The significantly lower distal CMAP amplitude of the tibial nerve and SNAP amplitude of the sural nerve in patients with CMT reflect the length-dependent axonal degeneration typical of this condition, as also confirmed in our cohort. While the motor conduction velocity (MCV) of the tibial nerve and sensory conduction velocity (SCV) of the sural nerve were also lower in CMT, this is likely attributable to reduced amplitude (Tankisi H et al., 2007). It is also important to note that in many cases, these nerves cannot be reliably measured due to undetectable responses, limiting their diagnostic utility. Although these findings further illustrate the pathophysiological differences between CIDP and CMT, none of these parameters outperformed the T/U CMAP amplitude ratio in terms of diagnostic accuracy, as shown by their lower AUC values in the ROC analysis.

The T/U CMAP amplitude ratio, measured in both the lower distal tibial nerve and the upper distal ulnar nerve, is likely reflective of the contrasting pathophysiological processes between CIDP and CMT. Moreover, the T/U CMAP amplitude ratio is calculated solely based on the distal CMAP amplitudes of the ulnar and tibial nerves. In our cohort, we also encountered cases initially labeled as CIDP in which overinterpretation of NCS findings—such as conduction block or temporal dispersion—likely contributed to misdiagnosis; prior reports have identified this as a major cause of CIDP misclassification (Allen et al., 2018). The T/U CMAP amplitude ratio is less vulnerable to such pitfalls and offers a simple, reliable readout. Indeed, even in these cases, the T/U CMAP amplitude ratio clearly distinguished the groups.

Our ROC analysis showed that the T/U CMAP amplitude ratio was the most effective parameter for distinguishing CIDP, with an AUC value of 0.95, in all patients with genetically confirmed CMT. At a cutoff value of 0.385, the ratio achieved a sensitivity of 95.5 % and a specificity of 85.5 %, indicating that the values above this threshold strongly indicate CIDP and lower values favor a CMT diagnosis. In addition, similar results were obtained in the subgroup analyses comparing demyelinating CIDP with demyelinating CMT and distal CIDP with CMT. In the comparison of subtypes with slow nerve conduction velocity, the length-dependent nature of CMT did not change. Although distal CIDP is clinically characterized by predominantly distal involvement, both proximal and distal segments are similarly affected in NCS. Based on these findings, the T/U CMAP amplitude ratio is a highly reliable electrophysiological marker with excellent diagnostic accuracy. Its high sensitivity ensures the effective identification of patients with CIDP. Meanwhile, its specificity helps reduce false-positive results. Hence, it can be a valuable tool for clinical decision-making.

Our study had several limitations. First, the retrospective design resulted in missing data, especially in regard to EDX parameter applications. Secondly, the number of patients differed substantially between the CIDP and CMT groups (44 vs 365), which may have influenced the statistical power to detect group differences. In addition, as this was a single-center study conducted in Japanese patients, the findings may not be fully generalizable to populations in other ethnic backgrounds. Further studies involving larger and more diverse cohorts are warranted to validate and extend our observations. Third, an evaluation of patients with undetectable ulnar nerve CMAP, such as those with severe neuropathy or ulnar neuropathy at the compression sites, could not be performed. Nevertheless, the electrophysiologic parameter of the T/U CMAP amplitude ratio applied in this study could also be gauged based on its robustness, given that patients with genetically confirmed CMT were included in the data for comparison with CIDP.

## 5 Conclusion

The T/U CMAP amplitude ratio is a simple electrophysiological index of length-dependent neuropathy. Its consistency across diverse CMT genotypes supports its use as a physiology-based triage tool to prioritize genetic testing and minimize prolonged, unnecessary immunotherapy. Considering its simplicity and reliability, this parameter has significant potential for clinical application, which enhances diagnostic accuracy in both acquired and inherited neuropathies and enables timely, targeted patient management.

## Authors contribution

Chikashi Yano, Tomonori Nakamura, and Kimiyoshi Arimura conceptualized and designed the study. Masahiro Ando, Yujiro Higuchi, Jun-Hui Yuan, Akiko Yoshimura, Akihiro Hashiguchi, Raymond L. Rosales, and Hiroshi Takashima contributed to the data analysis and interpretation. Takahiro Hobara, Fumikazu Kojima, Yu Hiramatsu, Satoshi Nozuma, and Yusuke Sakiyama were involved in the clinical data analysis. Chikashi Yano drafted the original manuscript, and all authors reviewed and approved the final version.

## Declaration of Competing Interest

The authors declare that they have no known competing financial interests or personal relationships that could have appeared to influence the work reported in this paper.

## References

[b0005] Allen J.A., Lewis R.A. (2015). CIDP diagnostic pitfalls and perception of treatment benefit. Neurology.

[b0010] Allen J.A., Ney J., Lewis R.A. (2018). Electrodiagnostic errors contribute to chronic inflammatory demyelinating polyneuropathy misdiagnosis. Muscle Nerve.

[b0015] Ando M., Higuchi Y., Yuan J., Yoshimura A., Taniguchi T., Kojima F. (2022). Comprehensive Genetic Analyses of Inherited Peripheral Neuropathies in Japan: Making Early Diagnosis Possible. Biomedicines.

[b0020] Attarian S., Azulay J.P., Boucraut J., Escande N., Pouget J. (2001). Terminal latency index and modified F ratio in distinction of chronic demyelinating neuropathies. Clin. Neurophysiol..

[b0025] Bouchard C., Lacroix C., Plante V., Adams D., Chedru F., Guglielmi J.M. (1999). Clinicopathologic findings and prognosis of chronic inflammatory demyelinating polyneuropathy. Neurology.

[b0030] Brown W.F., Snow R. (1991). Patterns and severity of conduction abnormalities in Guillain-Barre syndrome. J. Neurol. Neurosurg. Psychiatry.

[b0035] Campagnolo M., Taioli F., Cacciavillani M., Ruiz M., Luigetti M., Salvalaggio A. (2020). Sporadic hereditary neuropathies misdiagnosed as chronic inflammatory demyelinating polyradiculoneuropathy: Pitfalls and red flags. J. Peripher. Nerv. Syst..

[b0040] Ginsberg L., Malik O., Kenton A.R., Sharp D., Muddle J.R., Davis M.B. (2004). Coexistent hereditary and inflammatory neuropathy. Brain.

[b0045] Gutierrez A., England J.D., Sumner A.J., Ferer S., Warner L.E., Lupski J.R. (2000). Unusual electrophysiological findings in X-linked dominant Charcot-Marie-Tooth disease. Muscle Nerve.

[b0050] Hauw F., Fargeot G., Adams D., Attarian S., Cauquil C., Chanson J.B. (2021). Charcot-Marie-Tooth disease misdiagnosed as chronic inflammatory demyelinating polyradiculoneuropathy: An international multicentric retrospective study. Eur. J. Neurol..

[b0055] Kang J.H., Kim H.J., Lee E.R. (2013). Electrophysiological evaluation of chronic inflammatory demyelinating polyneuropathy and charcot-marie-tooth type 1: dispersion and correlation analysis. J. Phys. Ther. Sci..

[b0060] Kimura J. Electrodiagnosis in diseases of nerve and muscle : principles and practice. 3 ed: Oxford University Press, 2001. xxviii, 991 p. p.

[b0065] Kishi M., Sakakibara R., Takahashi O., Nakamura H., Tateno F., Tsuyusaki Y. (2017). Seiza-induced neuropathy: an occupational peroneal neuropathy in a Japanese lady. Neurol. Sci..

[b0070] Kishimoto S., Sasaki H., Kurisu S., Ogawa K., Matsuno S., Furuta H. (2021). Bilateral atrophy of the extensor digitorum brevis muscle might be a useful sign for diagnosing diabetic polyneuropathy in Japanese men who do not sit in the traditional “seiza” style. J Diabetes Investig.

[b0075] Kitaoji T., Noto Y.I., Kojima Y., Tsuji Y., Kitani-Morii F., Mizuno T. (2023). Compound muscle action potential duration ratio for differentiation between Charcot-Marie-Tooth disease and CIDP. Clin. Neurophysiol..

[b0080] Lewis R.A., Sumner A.J. (1982). The electrodiagnostic distinctions between chronic familial and acquired demyelinative neuropathies. Neurology.

[b0085] Marques W., Funayama C.A., Secchin J.B., Lourenco C.M., Gouvea S.P., Marques V.D. (2010). Coexistence of two chronic neuropathies in a young child: Charcot-Marie-Tooth disease type 1A and chronic inflammatory demyelinating polyneuropathy. Muscle Nerve.

[b0090] Michell A.W., Laura M., Blake J., Lunn M.P., Cox A., Gibbons V.S. (2009). GJB1 gene mutations in suspected inflammatory demyelinating neuropathies not responding to treatment. J. Neurol. Neurosurg. Psychiatry.

[b0095] Miki Y., Tomiyama M., Haga R., Nishijima H., Suzuki C., Kurihara A. (2013). A family with IVIg-responsive Charcot-Marie-Tooth disease. J. Neurol..

[b0100] Murphy S.M., Laura M., Blake J., Polke J., Bremner F., Reilly M.M. (2011). Conduction block and tonic pupils in Charcot-Marie-Tooth disease caused by a myelin protein zero p.Ile112Thr mutation. Neuromuscul. Disord..

[b0105] Street V.A., Meekins G., Lipe H.P., Seltzer W.K., Carter G.T., Kraft G.H. (2002). Charcot-Marie-Tooth neuropathy: clinical phenotypes of four novel mutations in the MPZ and Cx 32 genes. Neuromuscul. Disord..

[b0110] Tabaraud F., Lagrange E., Sindou P., Vandenberghe A., Levy N., Vallat J.M. (1999). Demyelinating X-linked Charcot-Marie-Tooth disease: unusual electrophysiological findings. Muscle Nerve.

[b0115] Tankisi H., Pugdahl K., Johnsen B., Fuglsang-Frederiksen A. (2007). Correlations of nerve conduction measures in axonal and demyelinating polyneuropathies. Clin. Neurophysiol..

[b0120] Vaeth S., Andersen H., Christensen R., Jensen U.B. (2021). A Search for Undiagnosed Charcot-Marie-Tooth Disease Among Patients Registered with Unspecified Polyneuropathy in the Danish National Patient Registry. Clin. Epidemiol..

[b0125] Van den Bergh P.Y.K., van Doorn P.A., Hadden R.D.M., Avau B., Vankrunkelsven P., Allen J.A. (2021). European Academy of Neurology/Peripheral Nerve Society guideline on diagnosis and treatment of chronic inflammatory demyelinating polyradiculoneuropathy: Report of a joint Task Force-Second revision. Eur. J. Neurol..

[b0130] Verhamme C., van Schaik I.N., Koelman J.H., de Haan R.J., Vermeulen M., de Visser M. (2004). Clinical disease severity and axonal dysfunction in hereditary motor and sensory neuropathy Ia. J. Neurol..

[b0135] Watanabe M., Yamamoto N., Ohkoshi N., Nagata H., Kohno Y., Hayashi A. (2002). Corticosteroid- responsive asymmetric neuropathy with a myelin protein zero gene mutation. Neurology.

[b0140] Yoshimura A., Yuan J.H., Hashiguchi A., Ando M., Higuchi Y., Nakamura T. (2019). Genetic profile and onset features of 1005 patients with Charcot-Marie-Tooth disease in Japan. J. Neurol. Neurosurg. Psychiatry.

