# Supplementary figures and images for: Tibial to ulnar nerve amplitude ratio as a marker of length-dependent neuropathy
Source: Clin Neurophysiol Pract. 2025 Oct 25;10:499–506. doi: 10.1016/j.cnp.2025.10.006 (PMC12648492; doi:10.1016/j.cnp.2025.10.006)

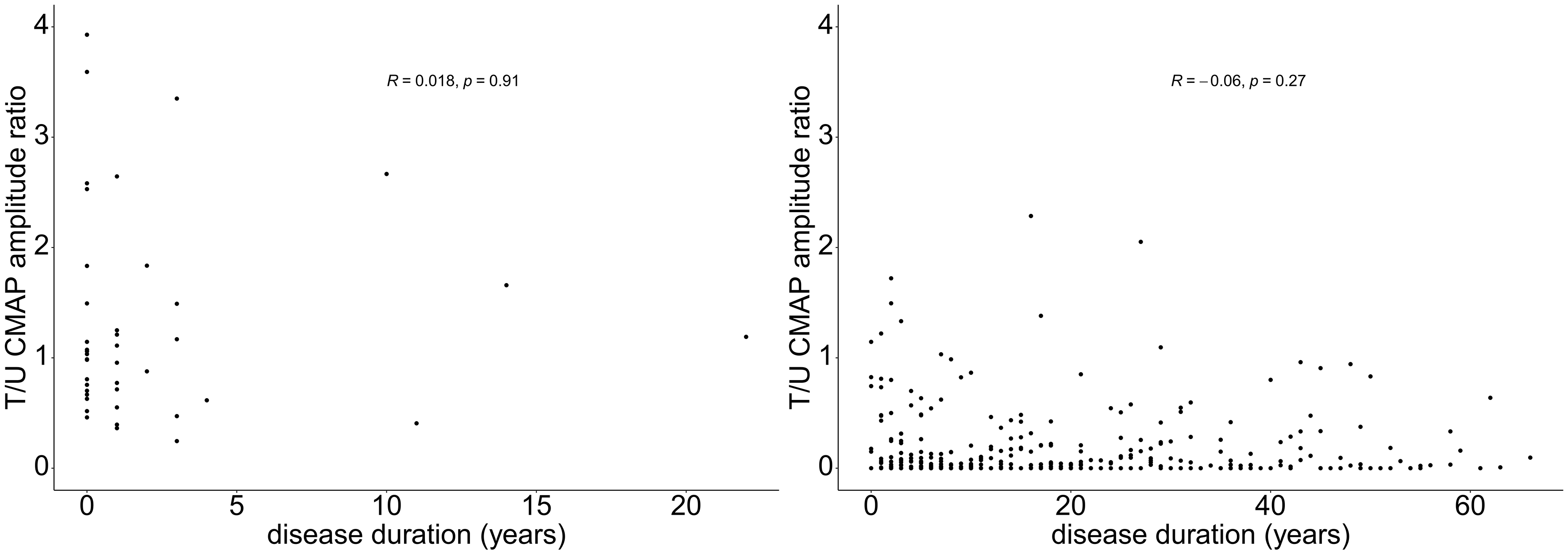

Supplement: Supplementary Fig. 1 [file mmc1.jpg]
