# Supplementary material for: Tibial to ulnar nerve amplitude ratio as a marker of length-dependent neuropathy
Source: Clin Neurophysiol Pract. 2025 Oct 25;10:499–506. doi: 10.1016/j.cnp.2025.10.006 (PMC12648492; doi:10.1016/j.cnp.2025.10.006)
Supplement: Supplementary Data 1 [file mmc2.docx]

Table S1. CMT patients initially diagnosed as CIDP

| Sex | Onset | Age | gene | Immunotherapy | Response to immunotherapy | Time to  diagnosis of CMT | Comment | Family history |
| --- | --- | --- | --- | --- | --- | --- | --- | --- |
| F | 68 | 75 | MME | IVIG | No response | 7 yrs |  | No |
| M | 42 | 44 | MME | IVIG | No response | 2 yrs |  | No |
| M | 42 | 52 | GJB1 | IVIG | No response | 11 yrs | Albuminocytologic dissociation (CSF 1 cell/μL; protein 79 mg/dL) | No |
| M | 46 | 49 | MPZ | IVIG | No response | 1.5 yrs |  | No |
| M | 45 | 51 | MME | IVIG; CS | No response | 5 yrs | Albuminocytologic dissociation | Yes |
| M | 23 | 27 | GJB1 | IVIG; DEX | No response | 4 yrs |  | Yes |
| M | 57 | 67 | MPZ | None | Not applicable | Not documented | Albuminocytologic dissociation (CSF 1 cell/μL; protein 67 mg/dL) | Yes |
| F | 61 | 67 | MPZ | IVIG; IVMP | No response | Not documented |  | Yes |
| M | 52 | 59 | MME | None | Not applicable | Not documented | Temporal dispersion of the tibial nerve compound muscle action potential | No |
| M | 7 | 38 | GJB1 | None | Not applicable | Not documented | Conduction block on NCS | Yes |
| F | 55 | 70 | MME | IVIG | No response | 15 yrs |  | Yes |
| F | 69 | 74 | MME | IVIG | Subjective improvement | 3 mo |  | No |
| M | 48 | 59 | GJB1 | IVIG (4) | No response | 2 yrs | Temporal dispersion on NCS; gadolinium enhancement of the lumbar plexus on MRI | No |
| M | 22 | 29 | GJB1 | IVIG; PE; IVMP | Subjective improvement | 7 yrs |  | Yes |
| F | 74 | 75 | MPZ | IVIG; IVMP | No response | 1 yr |  | No |
| F | 43 | 44 | MPZ | IVIG | No response | 1 yr |  | No |
| M | 55 | 68 | MME | IVMP | No response | 13 yrs |  | No |
| F | 62 | 65 | MME | IVIG | Subjective improvement | 6 yrs |  | No |
| F | 46 | 66 | MPZ | IVIG | Unknown | 4 mo |  | Yes |
| F | 36 | 51 | MPZ | IVIG (2) | No response | 1 yr | Muscle biopsy: group fiber atrophy; nerve biopsy: reduced large myelinated fibers, no onion-bulb formation | Yes |
| M | 18 | 68 | MFN2 | IVIG | No response | 5 mo | Albuminocytologic dissociation (CSF 1 cell/μL; protein 47 mg/dL) | Yes |
| M | 15 | 73 | MFN2 | None | Not applicable | Not documented |  | No |
| M | 57 | 59 | MPZ | IVIG (4) | Subjective improvement | 2 yrs |  | No |
| M | 66 | 73 | MPZ | IVIG | Subjective improvement | 2 yrs | Demyelinating features on NCS | Yes |
| M | 66 | 69 | GJB1 | IVIG | No response | 2 yrs |  | Yes |
| F | 54 | 64 | MME | IVIG; IVMP; CS | Objective improvement | 6 yrs | Temporal dispersion on NCS | Yes |
| M | 36 | 49 | MME | IVIG (5); PE (2) | No response | 7 yrs |  | No |

CMT, Charcot–Marie–Tooth disease; CIDP, chronic inflammatory demyelinating polyradiculoneuropathy; M, male; F, female; IVIG, intravenous immunoglobulin; CS, corticosteroids; DEX, dexamethasone; IVMP, Intravenous methylprednisolone; PE, plasma exchange; NCS, nerve conduction studies; MRI, magnetic resonance imaging; CSF, cerebrospinal fluid.
